# Carvacrol and linalool co-loaded in β-cyclodextrin-grafted chitosan nanoparticles as sustainable biopesticide aiming pest control

**DOI:** 10.1038/s41598-018-26043-x

**Published:** 2018-05-16

**Authors:** Estefânia V. R. Campos, Patrícia L. F. Proença, Jhones L. Oliveira, Anderson E. S. Pereira, Lígia Nunes de Morais Ribeiro, Fabrício O. Fernandes, Kelly C. Gonçalves, Ricardo A. Polanczyk, Tatiane Pasquoto-Stigliani, Renata Lima, Cirano C. Melville, Jaqueline F. Della Vechia, Daniel J. Andrade, Leonardo F. Fraceto

**Affiliations:** 1 São Paulo State University – UNESP, Institute of Science and Technology, Sorocaba, SP Brazil; 2 Department of Biochemistry and Tissue Biology, State University of Campinas, Campinas, SP Brazil; 3 São Paulo State University – UNESP, College of Agricultural and Veterinary Sciences, Department of Crop Protection, Jaboticabal, SP Brazil; 4 Department of Biotechnology, University of Sorocaba, Sorocaba, Brazil

## Abstract

Pesticides are the main tactics for pest control because they reduce the pest population very fast and their efficiency does not depend on abiotic factors. However, the indiscriminate use of these substances can speed up the development of resistant populations and causing environmental contamination. Therefore, alternative methods of pest control are sought, such as the use of botanical compounds. Nanoencapsulation of volatile compounds has been shown to be an important tool that can be used to overcome the lack of stability of these compounds. In this work, we describe the preparation and characterization of chitosan nanoparticles functionalized with β-cyclodextrin containing carvacrol and linalool. The toxicity and biological activity were evaluated. Decreases of toxicity were observed when the compounds were nanoencapsulated. The nanoparticles presented insecticidal activity against the species *Helicoverpa armigera* (corn earworm) and *Tetranychus urticae* (spider mite). In addition, repellent activity and reduction in oviposition were observed for the mites.

## Introduction

Approximately 67,000 species of organisms are known to attack crops, among which insects and mites are among the most virulent invasive species and are responsible for reducing global agricultural production by about 10 to 16%. According to Bradshaw *et al*.^1^, ten insect species alone are responsible for annual losses of US$ 77 billion per year worldwide, and this number could increase by around 18% by 2050, due to climate change. In Brazilian agriculture, damage caused by insects results in a loss of 25 million tons of food, fiber, and biofuels annually^2^.

*Helicoverpa armigera* (Hübner) (Lepidoptera: Noctuidae), world bollworm, is considered the most important agricultural insect pest worldwide^3^. It is polyphagous and has a wide geographical distribution, being found in Africa, Asia, Australia, China, Europe, and Oceania^4^, as well as in some South American countries^5,6^. In 2015, Inspection Service of the United States Department of Agriculture detected *H*. *armigera* in the continental United States^7^. In addition to polyphagia, the success of *H*. *armigera* as a pest is due to its high reproduction rate and facultative diapause, which favor increased spatial distribution among hosts^8^, as well as the capacity to adapt to adverse conditions of humidity and temperature^9^, resistance to the routinely used insecticides^10,11^ and resistance to plants expressing the Bt protein^7,12,13^.

Another agriculturally important species, due to the extent of the damage caused and its wide range of hosts, is the two-spotted spider mite *Tetranychus urticae* Koch (Acari: Tetranychidae)^14,15^. This is one of the most serious pests in world agriculture, which feeds on the leaves of the plants, causing damage to the chlorophyll and the formation of white patches on the leaves that increase over time. As a result, the production of carbohydrates by the plants decreases and consequently the plant growth is reduced^15^.

The application of pesticides is the tool most widely used worldwide for the control of *H*. *armigera* and *T*. *urticae*^16,17^. However, due to the indiscriminate and uncontrolled use, especially carbamates, cyclodienes, organophosphates, and pyrethroids, many cases of resistance have been reported, resulting in failure to effectively control pest populations^18–21^. In order to delay the development of resistant populations, research is needed to develop better systems for pest control. One option is the use of botanical insecticides that can provide pest control, while reducing environmental damage and effects on nontarget organisms^22–25^.

Carvacrol (2-methyl-5-(1-methylethyl) phenol) (CVC) is a phenolic monoterpene found in the essential oils of thyme (*Thymus vulgaris*), marjoram (*Origanum majorana*), oregano (*Origanum vulgari* L.), pepperwort (*Lepidium* sp.), and Alaskan yellow cedar (*Callitropsis nootkatensis* (D. Don) Oerst. ex D. P. Little). This compound presents antimicrobial activity^26^ and for this reason is widely used as a food additive. It is also highly toxic to many invertebrates, including mites, insects, and nematodes. In insects, the action of carvacrol is due to the noncompetitive inhibition of acetylcholinesterase^27^ by interaction with nicotinic acetylcholine receptors^28^. Linalool (3,7-dimethyl-1,6-octadien-3-ol) (LNL) is an alcoholic monoterpene found in the essential oils of rosewood (*Aniba rosaeodora* Ducke), sacaca (*Croton cajucara* Benth), and basil (*Ocimum basilicum* L.). It is known to have antifungal, antimicrobial, and repellent activities^29^. It acts on the nervous system of insects by the reversible inhibition of acetylcholinesterase^30^.

Although these monoterpenes have been extensively studied for pest control in agriculture^22,31,32^, their large-scale use is limited due to their low aqueous solubility, high volatility, and fast degradation when exposed to the environment. The low persistence of these compounds means that a greater number of applications may often be required in order to achieve the desired effect^33,34^. One way to reduce the degradation of these compounds by environmental factors is to incorporate them into nanostructured systems^35,36^.

The substances that constitute a particular essential oil can have antagonistic, additive, or synergistic effects in pest control. Some studies have found that essential oils have more pronounced effects on pests, compared to the effect of the major component alone^37^, suggesting that minor components present in essential oils are essential for enhancement of the effects on target organisms. Hence, the combination of substances present in the same essential oil and/or essential oils of different species may affect the pest in different ways, and can therefore be used as a strategy for pest control that delay the risk of the emergence of resistant pests.

Polysaccharides are widely used in sustained release formulations with applications in a variety of areas, including agriculture. Polysaccharides are molecules that are widely distributed in nature, composed of monosaccharides linked by glycosidic bonds. Advantages of these compounds include low cost and high availability, which can facilitate large-scale production^38^. Chitosan is a polymer that is soluble in acidic media and is highly hydrophilic due to the presence of numerous hydroxyl and amine groups in its structure, making it versatile for use in the production of different carrier systems^39,40^. These reactive groups enable reaction with various different molecules^40^, such as β-cyclodextrin, a cyclic polysaccharide composed of glucose units (α-D-glucopyranose) linked by α-(1,4) bonds, which has a high capacity to form inclusion complexes with hydrophobic molecules^41^. Hence, the functionalization of a chitosan skeleton with cyclodextrin molecules can generate a carrier that possesses the cumulative effects of the inclusion, size specificity, and transport properties of cyclodextrin, together with the sustained release property of chitosan^42^. Recently we have been published a study with the full characterization of the functionalization of the chitosan glycol with β-cyclodextrin as well as the interaction of carvacrol and linalool with β-cyclodextrins^43^. Also in this study we have been prepared chitosan grafted β-cyclodextrin and tripolyphosphate loaded with carvacrol or linalool as well as investigated the physicochemical properties of the nanoparticles as well the biological activity against spider mite *Tetranychus urticae*. The results showed that the nanoparticles had good colloidal characteristics and high encapsulation efficiencies for both carvacrol and linalool. In addition, these nanoparticles were effective in reduce *T. urticae* population through repellence, acaricidal and oviposition inhibition activities.

The objective of this work was to prepare and characterize nanoparticles of chitosan functionalized with cyclodextrin containing carvacrol and linalool using the emulsion/ionic gelation method. The average diameter, polydispersity index, and concentration of the particles were evaluated as a function of storage time (up to 60 days). Determination was made of the encapsulation efficiency of the nanoparticles and the *in vitro* release kinetics. Evaluation was also made of the cytotoxicity and phytotoxicity of the nanoparticles and their bioactivity against *H*. *armigera* and *T*. *urticae*. It should be highlighted that there have been no studies reported in the literature using this type of particle in association with the active agents considered in this study, which is aimed at future applications in sustainable approaches to agricultural management. The present study therefore opens perspectives for safer and more effective solutions employing compounds of botanical origin associated with nanotechnology for the control of pests in agriculture.

## Materials and Methods

### Materials

Carvacrol (98%), linalool (97%), β-cyclodextrin (MW: 1134.98 g/mol), chitosan glycol (≥60% titration; degree of polymerization ≥400), Gum Arabic (MW: ~250.000 g/mol), and Tween 80 were obtained from Sigma-Aldrich and were used without further purification. Acetonitrile and methanol (HPLC grade) were obtained from J.T. Baker.

### Preparation of functionalized chitosan/gum arabic nanoparticles (CDgCS/GA)

The functionalization of β-CD in the chitosan glycol skeleton was performed according to the procedure described by^44^. The preparation and characterization of the modified chitosan was previously described by Campos *et al*.^44^. Functionalized chitosan nanoparticles crosslinked with gum arabic were obtained using the method of Avadi *et al*.^45^. Firstly, the chitosan (1.5% m/v) was diluted in an aqueous solution containing 0.5% acetic acid and stirred overnight. Portions of 25 mg of each oil (carvacrol and linalool) and 150 mg of Tween 80 surfactant were then added to the chitosan solution, followed by emulsification using an Ultraturrax homogenizer (Ika) at 5000 rpm for 5 minutes. Next, a solution of gum arabic was added to the organic phase using a syringe and the crosslinking reaction was allowed to proceed for 15 minutes. This method essentially consists of ionic gelation between the opposing charges of chitosan (positive) and gum arabic (negative)^45^.

### Physico-chemical characterization of the nanoparticles

#### Mean diameter, polydispersity index, and zeta potential

The hydrodynamic diameter and polydispersity index of the nanoparticles with or without CVC and LNL were determined by the dynamic light scattering (DLS) technique. The zeta potential was determined by the microelectrophoresis technique. These measurements were performed following dilution of the nanoparticle suspensions 100-fold in deionized water, using a ZetaSizer Nano ZS 90 particle analyzer (Malvern Instruments) at a fixed angle of 90° and temperature of 25 °C.

### Determination of nanoparticle mean diameter and concentration by nanoparticle tracking analysis (NTA)

NTA analyses were performed with a NanoSight LM10 instrument, using a laser with wavelength of 532 nm (green), a CMOS camera, and NanoSight v. 3.1 software. The nanoparticle suspensions were diluted 40-fold before being transferred to the volumetric cell of the instrument and the analyses were performed in triplicate, each with five measurements for 60 seconds. In order to ensure that different particles were analyzed in each replicate, a volume greater than the cell capacity was injected, hence displacing the content analyzed.

### Encapsulation efficiency

The amounts of CVC and LNL associated with the nanoparticles were determined by the ultrafiltration/centrifugation technique. The concentrations of the compounds present in the ultrafiltrate were determined by HPLC. The percentage encapsulated in the nanoparticles was determined by the difference between the concentration added (100%) and the concentration present in the ultrafiltrate. The analytical curves used for quantification were as follows: CVC = 5.4666x − 0.065 (r^2^ = 0.9989); LNL = 1.2262x+ 1.6471 (r^2^ = 0.9990).

### Release kinetics and mathematical modeling

The release kinetics of CVC and LNL were determined according to the method described by Kim *et al*.^46^. Suspensions (2 mL) of nanoparticles containing CVC and LNL (at 1.25 mg/mL) were placed on dialysis membranes with exclusion pore size of 12–15 kDa. The dialysis membranes were then transferred to 100 mL of aqueous solution containing Tween 80 (1.5% v/v), with gentle shaking at 100 rpm on an orbital shaker, at 25 °C. At predetermined times, 1 mL aliquots were removed from the medium external to the dialysis membrane and the concentrations of CVC and LNL were determined by HPLC. In order to maintain the same volume of medium throughout the experiment, the volume withdrawn was replaced with aqueous Tween 80 solution. The mechanisms of release of CVC and LNL from the nanoparticles were evaluated using the zero order, first order, Higuchi, Hixson-Crowell, and Korsmeyer-Peppas mathematical models^47^.

### Morphological analysis

The morphologies of the different nanocarrier systems were analyzed by transmission electron microscopy (TEM) and atomic force microscopy (AFM). For the TEM analyses, the nanoparticle suspensions were diluted 1000 times and a small aliquot was dripped onto 200 mesh grids. After drying, contrast was provided by dripping a solution of 2% uranyl acetate onto the grids. After evaporation, the samples were examined using a Zeiss LEO 906 microscope operated at a voltage of 80 kV. For the AFM analyses, the nanoparticle suspensions were diluted 500-fold and then dripped onto a silicon plate. After drying, the samples were analyzed using a Nanosurf Easy Scan 2 Basic atomic force microscope (Nanosurf, Switzerland) in non-contact mode. The instrument was equipped with a TapAl-G cantilever (BudgetSensors, Bulgaria) operated at a scan rate of 90 Hz. The images (256 × 256 pixels, TIFF format) were captured in time mode and were analyzed using Gwyddion software.

### Cell viability cytotoxicity assays

The cytotoxic effects of the nanoformulations containing CVC and LNL, as well as the emulsified compounds, were evaluated by the WST-8 method (Cell Counting Kit-8, Sigma-Aldrich) applied to pulmonary (v79) and mouse fibroblast (Balb C-3T3) cell lines. The cells were seeded at a concentration of 1 × 10^5^ cells in 96-well plates with supplemented DMEM medium and were incubated at 37 °C in an oven with 5% CO_2_ for a period of 24 hours. The cells were then treated for 24 hours with the emulsified CVC and LNL and the nanoparticles with or without the bioactive compounds at concentrations in the range 0.2–1.6 mM. After this time, 10 μL of 2-(2-methoxy-4-nitrophenyl)-3-(4-nitrophenyl)-5-(2,4-disulfophenyl) (WST-8) was added to each well and the plates were incubated for 4 hours. Cell viability was assessed by the reduction of WST-8 to formazan by the viable cells. The absorbance of the solution in each well was then measured at 450 nm using a plate reader (Multiskan MS, Labsystems) and the percentage of viable cells was calculated.

### Phytotoxicity assays

The effects of the nanoencapsulated and emulsified CVC and LNL on plants were evaluated using pre- and post-emergence treatments of maize (*Zea mays* L.) seedlings. For the pre-emergence treatment, the seeds were treated for 1 hour with the control nanoparticles formulation (NP), nanoparticles containing CVC and LNL (NP_C + L), emulsified CVC and LNL (C + L + Tween 1.5%), and surfactant solution (1.5% Tween). The bioactive compounds were used at concentrations of 0.05, 0.25, and 2.5 mg/mL. After treatment, the seeds were planted in 9.3 cm pots filled with 600 g of Carolina Soil substrate (sphagnum peat, expanded vermiculite, dolomitic limestone, agricultural chalk, and NPK fertilizer). For the post-emergence treatments, 5 mL volumes of the formulations were sprayed onto the pots 15 days after germination, using the same concentrations employed in the pre-emergence assays. The pots were kept in a greenhouse under natural conditions of illumination and temperature. After 21 days, the plants were collected and the lengths of the shoots and roots were measured. All the treatments were performed in triplicate (n = 3)^48^.

Chlorophylls A and B and carotenoids were also determined for each treatment. For this, 5 mm^2^ disks were removed from the seedling leaves and placed in tubes containing 2 mL of DMSO. The tubes were wrapped in aluminum foil to protect them from light and were stored in a refrigerator for 24 hours, followed by measurements using a UV-Vis spectrophotometer (Cary 50, Varian) at wavelengths of 665, 649, and 480 nm to determine chlorophyll A, chlorophyll B, and carotenoids, respectively^49^.

### Bioactivity assays

#### *Helicoverpa armigera*

The insecticidal bioactivity bioassays were performed at the Microbial Control of Pest Arthropods Laboratory (UNESP/FCAV, Jaboticabal campus). Aliquots of 400 μL of the formulations of nanoparticles containing CVC and LNL (NP_C + L) and the emulsified CVC and LNL (C + L + Tween 1.5%), all at concentrations of 1.25 mg/mL of each active agent, were applied to the surfaces of artificial diet disks (4.8 cm^3^) arranged on acrylic plates (3.5 cm diameter). A control disk was treated with the same volume of sterilized distilled water. After allowing excess moisture to evaporate, five second instar larvae were placed on the diet disks, using five replicates. The plates were incubated in a biological oxygen demand (B.O.D) type incubator at 25 ± 1 °C and 70 ± 10% RH, with a photoperiod of 14 hours. The larval mortality was evaluated on the 7^th^ day. In addition, the sublethal effects of the formulations were evaluated by weighing the larvae 120 minutes after the start of the experiment and again 24 hours after reaching the pupal stage.

#### *Tetranychus urticae*

The bioassays with the species *T*. *urticae* were performed at the Acarology Laboratory (UNESP/FCAV, Jaboticabal campus). The mites were reared in a greenhouse on bean plants (*Phaseolus vulgaris*), in an area free from the application of agrochemicals in order to avoid the development of resistance by the mites. Evaluation was made of the insecticidal and repellent activities of the different formulations, as well as the effects on oviposition, using the nanoparticles containing CVC and LNL (NP_C + L) and the emulsified CVC and LNL (C + L + Tween 1.5%), all at concentrations of 1.25 mg/mL of each active agent. Disks 2.5 cm in diameter were cut from the leaves of the bean host plants. These disks (arenas) were placed in Petri dishes (9 × 2 cm) containing a humidified foam (height 1.0 cm) and a thin layer of hydrophilic cotton on top of the foam. The escape of the mites (and consequently the repellent activity) was evaluated using entomological glue applied around the arena. Two milliliters of the emulsified or nanoencapsulated CVC and LNL formulations were applied with a Potter spray tower calibrated at 4 lbf.in^−2^, corresponding to 1.56 mg cm^−2^ in each arena. After drying the leaves for approximately 2 hours, ten adult *T*. *urticae* females were transferred to each arena, with each treatment being repeated eight times and each repetition consisting of one arena, totaling 80 mites per treatment. The arenas were conditioned in a climate chamber at a temperature of 25 ± 1 °C, relative humidity of 60 ± 10%, and photoperiod of 12 hours. Quantification was made of the numbers of live and dead mites, mites adhered to the glue barrier, and the number of eggs, at times of 12, 24, 48, and 72 hours following transfer of the mites. The mite repellency percentage (% repellency) was calculated considering the total number of mites and the number adhered to the entomological glue barrier. The acaricidal effect percentage was calculated considering the total number of mites and the number of dead mites. The effect on oviposition was calculated considering the total number of eggs divided by the number of live mites.

### Data analysis

All the experiments were performed at least in triplicate and the results were expressed as the mean of three determinations ± standard deviation. Statistical analysis was performed with Graphpad Prism 6.0 software, using two-way ANOVA (p < 0.05 was considered significant).

## Results

In this work, a carrier system based on chitosan nanoparticles functionalized with β-cyclodextrin was developed to carry two botanical insecticides (carvacrol and linalool) together, in order to create an effective and environmentally safe system for the control of agricultural pests. These two naturally-occurring polymers were selected for use in the nanoparticulate systems due to their desirable characteristics including low toxicity, biocompatibility, and biodegradability.

For the development of the modified chitosan with β-CD used in this study we have been used a chitosan with high aqueous solubility (chitosan glycol – ≥60% titration; degree of polymerization ≥400) since the functionalization of cyclodextrin occurs in the amino groups, which are essential for the solubilization of this polymer and nanoparticle formation. The preparation of this hybrid polymer (chitosan and β-CD) and the characterization using different analytical techniques was extensively reported by Campos *et al*.^44^. In this previous study the preparation and characterization of inclusion complexes of β-cyclodextrin with CVC or LNL were also evaluated. The results showed a higher affinity of β-cyclodextrin for CVC when compared to LNL, which can be explained by the physico-chemical characteristics of the active ingredients (i.e lower aqueous solubility and lower vapor pressure of CVC when compared to the LNL). In this sense, these properties were capable of influencing the characteristics of the nanoparticles of chitosan crosslinked with tripolyphosphate loaded with CVC or LNL.

Here, we have been prepared chitosan functionalizated nanoparticles with β-CD and gum arabic co-loading CVC and LNL. The physico-chemical properties and colloidal stability of the control nanoparticles (NP) and the nanoparticles containing carvacrol and linalool (NP_C + L) were evaluated as a function of storage time (up to 60 days). Table 1 summarizes the physico-chemical characteristics of the nanoparticles, including mean diameter, polydispersity index, zeta potential, particle concentration, and encapsulation efficiency measured at the start of the storage period.

**Table 1:** Characterization of the chitosan nanoparticles functionalized with β-CD containing CVC and LNL: mean diameter (MD) using the dynamic light scattering (DLS) and nanoparticle tracking analysis (NTA) techniques; polydispersity index (PDI); zeta potential (ZP); and concentration (CT). The values represent the means of three determinations.

Samples	MD (nm)		PDI	ZP (mV)	CT (10^12^ particles/mL)
	DLS	NTA			
NP	494.5 ± 16.8	310.8 ± 24.2	0.232 ± 0.07	13.1 ± 0.93	3.14 ± 0.138
NP_C + L	225.9 ± 5.23	194.7 ± 5.4	0.185 ± 0.02	19.3 ± 0.61	4.16 ± 0.478

Table 1 shows the colloidal properties of the nanoparticles at the start of the storage period. It can be seen that the mean diameter of the control nanoparticles (NP) determined by both techniques, as well as the polydispersity index, were higher than the values obtained for the nanoparticles containing the bioactive agents (NP_C + L), indicating that the addition of the compounds resulted in the formation of a less polydispersed system. Furthermore, the nanoparticles containing CVC and LNL presented a higher zeta potential (19.3 ± 0.61 mV), compared to the control nanoparticles (13.1 ± 0.93 mV). After 30 days of storage, there were significant increases in the mean diameter and polydispersity index of the control nanoparticles (NP), while the nanoparticles containing CVC and LNL (NP_C + L) showed no significant changes for any of the parameters analyzed (data not shown).

The initial concentration of the control nanoparticles was 3.14 ± 0.138 × 10^12^ particles/mL, with a decrease after 30 days of storage, in agreement with the increases in the mean diameter and the polydispersity index, indicative of the formation of aggregates in the control nanoformulation. However, for the nanoparticles containing CVC and LNL, there was a significant and progressive increase of the particle concentration. This phenomenon could indicate that over time, aggregates initially present in the formulation were broken down, hence causing the number of particles to increase.

The encapsulation efficiency was also used to investigate the stability of the nanoformulations containing carvacrol and linalool. The initial encapsulation efficiencies were 93.9 ± 0.58% for CVC and 86.9 ± 0.9% for LNL. These high encapsulation efficiencies could be explained by the high hydrophobicity of these compounds and their consequent strong affinity for the nanoparticle nucleus or the hydrophobic cavity of the cyclodextrins present in these systems. The affinity constants of CVC and LNL for the hydrophobic cavity of the β-CD at 25 °C were 157.8 ± 0.32 M^−1^ and 178.6 ± 1.01 M^−1^, respectively, as determined experimentally. In addition, the greater efficiency of CVC encapsulation, compared to LNL, could be explained by the higher partition coefficient (3.64) and lower aqueous solubility of CVC (0.11 g/L) than LNL (1.589 g/L).

Similar results were found by Hosseini *et al*.^50^, who prepared nanoparticles containing different mass ratios of chitosan and oregano essential oil using a two-step procedure employing an oil-in-water emulsion and ionic gelation of chitosan with sodium tripolyphosphate (TPP). An increase of the chitosan:essential oil ratio resulted in a higher mean diameter and a lower encapsulation efficiency. The mean nanoparticle diameter ranged from 309.8 ± 8.3 to 402.2 ± 10.7 nm and the encapsulation efficiency ranged from 21.1% to 47.7%. The highest encapsulation efficiency was observed for a nanoformulation prepared using a chitosan:essential oil ratio of 1:0.1(w/w). Lopez *et al*.^51^ studied different types of microencapsulated linalool, including the use of alginate/chitosan beads produced by the coacervation method. Compared to other carriers studied, the beads presented a low encapsulation yield (40%) and the mean diameter was around 1.8 ± 0.3 mm.

It should be noted that although the literature includes several studies concerning the preparation and characterization of carrier systems for botanical insecticides/repellents^52,53^, these studies did not determine the colloidal stability of the systems as a function of time. An additional novelty of the present work is the use of a combination of two botanical pesticides in the same nanoparticulate system, with the aim of improving the effectiveness of the system^54^.

### Release kinetics

**Figure 1:**
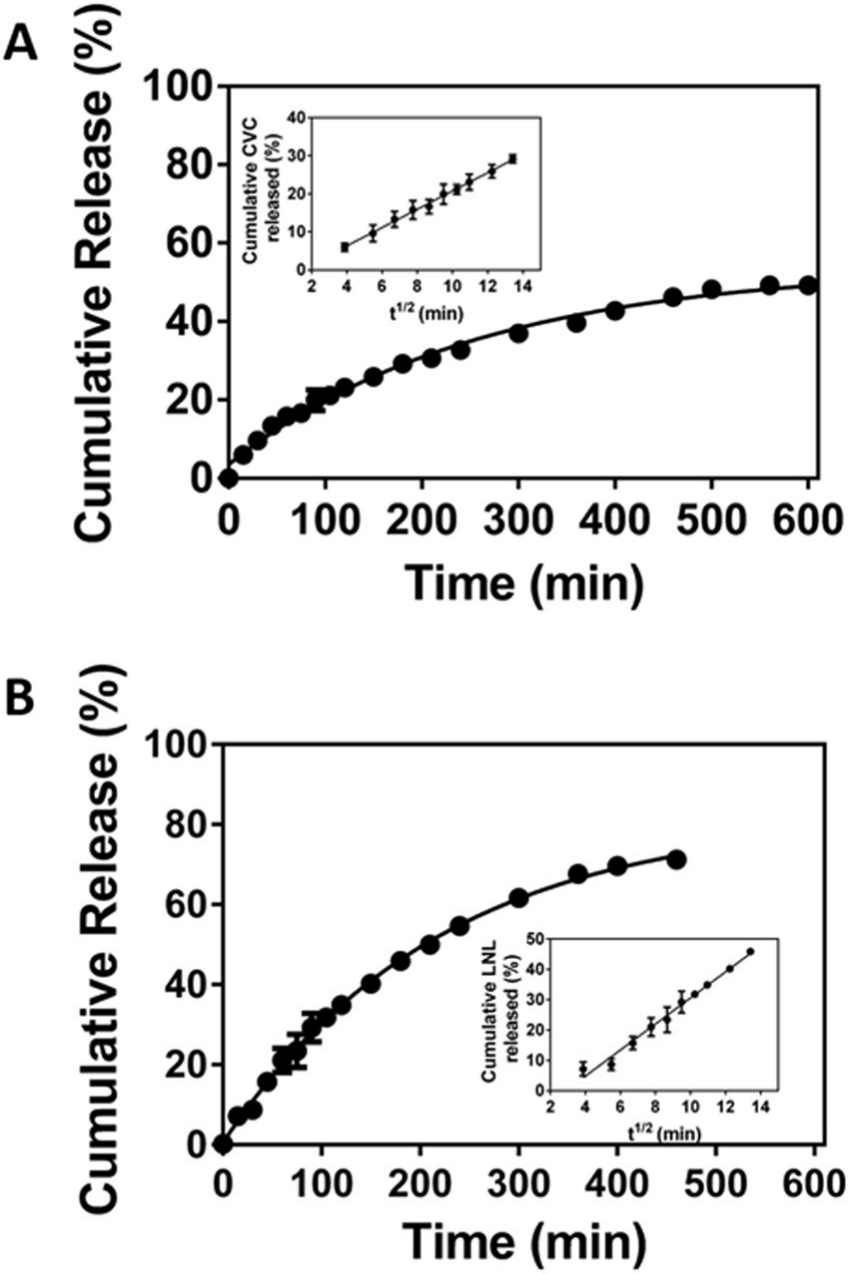
Kinetics of release of carvacrol (**A**) and linalool (**B**) from the chitosan nanoparticles functionalized with β-cyclodextrin. The release assay was performed by the dialysis membrane bag method at room temperature (25 °C), in Tween 80 solution (1.5% v/v). The analyses were performed in triplicate and quantified by HPLC. The inserts show the fits of the Higuchi mathematical model for the release of the compounds.

The release kinetics were investigated in order to understand the mechanisms of release of carvacrol and linalool from the chitosan nanoparticles functionalized with β-cyclodextrin. Figure 1A shows the profile of release of carvacrol from the chitosan/gum arabic nanoparticles, revealing substantial release in the first 60 minutes, followed by more gradual release. The faster initial release could be attributed to loss of the CVC molecules adsorbed on the surfaces of the nanoparticles or encapsulated near the surface^55^. After 600 minutes, carvacrol release reached 49.2 ± 0.45% of the total amount present in the nanoparticles. Rapid release was also observed for linalool (Fig. 1B), with over 20% of the compound being released within the first hour and 50% after 210 minutes. Maximum release of 71.2 ± 0.76% of the LNL was observed after 460 minutes.

Rapid initial release followed by more sustained release was also observed by Hosseini *et al*.^50^ for the essential oil of oregano. Nanoparticles prepared using a chitosan:oregano oil ratio of 1:0.1 (m/m) showed release of approximately 82% of the oil associated with the nanoparticles within the first three hours. In other work, carvacrol encapsulated in nanoparticles composed of the synthetic polymer D,L-lactide-co-glycolide (PLGA) showed an initial fast release (burst effect), followed by slower release. After three hours, 60% of the carvacrol contained in the nanospheres was released, with almost all of the compound (95%) contained in the nanoparticles being released after 24 hours^56^.

The profiles of release of the bioactive agents from the nanoparticles were analyzed by applying the zero order, first order, Higuchi, Korsmeyer-Peppas, and Hixson-Crowell mathematical models. Linear regression was used to calculate the values of the release constants (k) and the correlation coefficients (r). The results are summarized in Table 2.

**Table 2:** Parameter values obtained following application of the zero order, first order, Higuchi, Korsmeyer-Peppas, and Hixson-Crowell mathematical models to the curves for the release of CVC and LNL from the chitosan nanoparticles.

Parameter	**Zero order**	**First order**	**Higuchi**	**Korsmeyer-Peppas**	**Hixson-Crowell**
**NP_CVC**					
Release constant (k)	0.135 min^−1^	3.6 × 10^−3^ min^−1^	**2**.**4 min**^**−1/2**^	0.63 min^−1^	2.3 × 10^−3^ min^−1^
Correlation coefficient (r)	0.9695	0.8467	**0**.**9966**	0.9944	0.9776
**NP_LNL**					
Release constant (k)	0.242 min^−1^	4.6 × 10^−3^ min^−1^	**4**.**29 min**^**−1/2**^	0.79 min^−1^	9.6 × 10^−3^ min^−1^
Correlation coefficient (r)	0.9779	0.8312	**0**.**9880**	0.9701	0.8821

The best fits to the release profiles of both CVC and LNL were obtained using the Higuchi mathematical model (Table 2, Fig. 1), indicating that the mechanism of release of CVC and LNL from the nanoparticles was mainly by diffusion following Fick’s law. The release assays showed that for the same period of time (460 minutes), there was greater release (1.5-fold higher) of linalool than carvacrol. This difference in the release rates was also confirmed by the release constant (k) values obtained for the two compounds using the Higuchi mathematical model (Table 2), which showed that the rate of release of LNL was 1.8-fold faster, compared to the release of CVC. Possible reasons for the faster release of LNL include its higher aqueous solubility (1.589 g/L) and lower partition coefficient (2.97), compared to CVC (values of 0.11 g/L and 3.64, respectively). Due to the higher solubility of LNL, the molecules were likely to be located closer to the surface, compared to CVC, resulting in faster release of LNL. Keawchaoon & Yoksan^53^ studied chitosan nanoparticles crosslinked with pentasodium tripolyphosphate for the sustained release of carvacrol. It was found that the release of CVC was faster at more acidic pH, and that at all the pH values studied, the release exhibited non-Fickian behavior involving diffusion and swelling of the polymer chains.

### Morphological analyses

**Figure 2:**
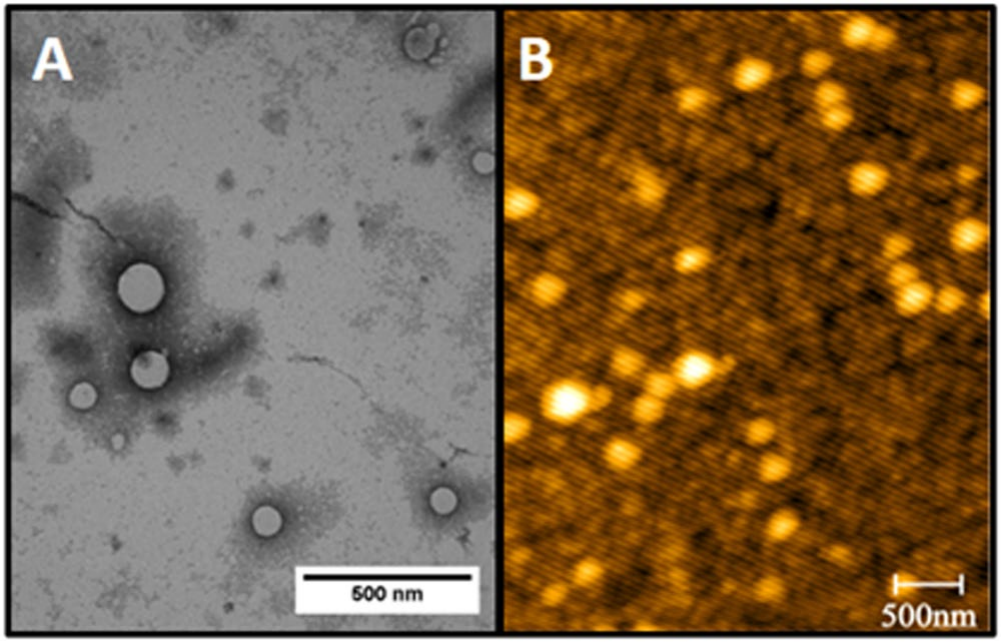
Micrographs obtained by transmission electron microscopy at 60x magnification (**A**) and micrographs obtained by atomic force microscopy (**B**) for the chitosan nanoparticles functionalized with β-cyclodextrin containing CVC and LNL.

Transmission electron microscopy (TEM) (Fig. 2A) and atomic force microscopy (AFM) (Fig. 2B) were used to investigate the morphology of the nanoparticles with encapsulated CVC and LNL. Both techniques revealed that the nanoparticles were spherical with smooth surfaces, with mean size distributions of 197.9 ± 16.8 and 194.6 ± 22.7 nm measured by TEM and AFM, respectively. These mean diameter values were smaller than those obtained by the DLS and NTA techniques, which could be explained by the absence of water, since the samples were dried prior to the microscopy analyses. Given that both of the bioactive compounds employed in this work are highly volatile, the absence of pores on the surface of the nanoparticles was a desirable feature, since it could help to protect the oils against degradation due to environmental factors Hosseini *et al*.^50^ also reported spherical and smooth morphology for chitosan/TPP nanoparticles containing CVC. Hou *et al*.^57^ functionalized N-maleoyl chitosan with β-cyclodextrin for loading with ketoprofen and found that the nanoparticles presented spherical morphology.

### Cell viability assays

**Figure 3:**
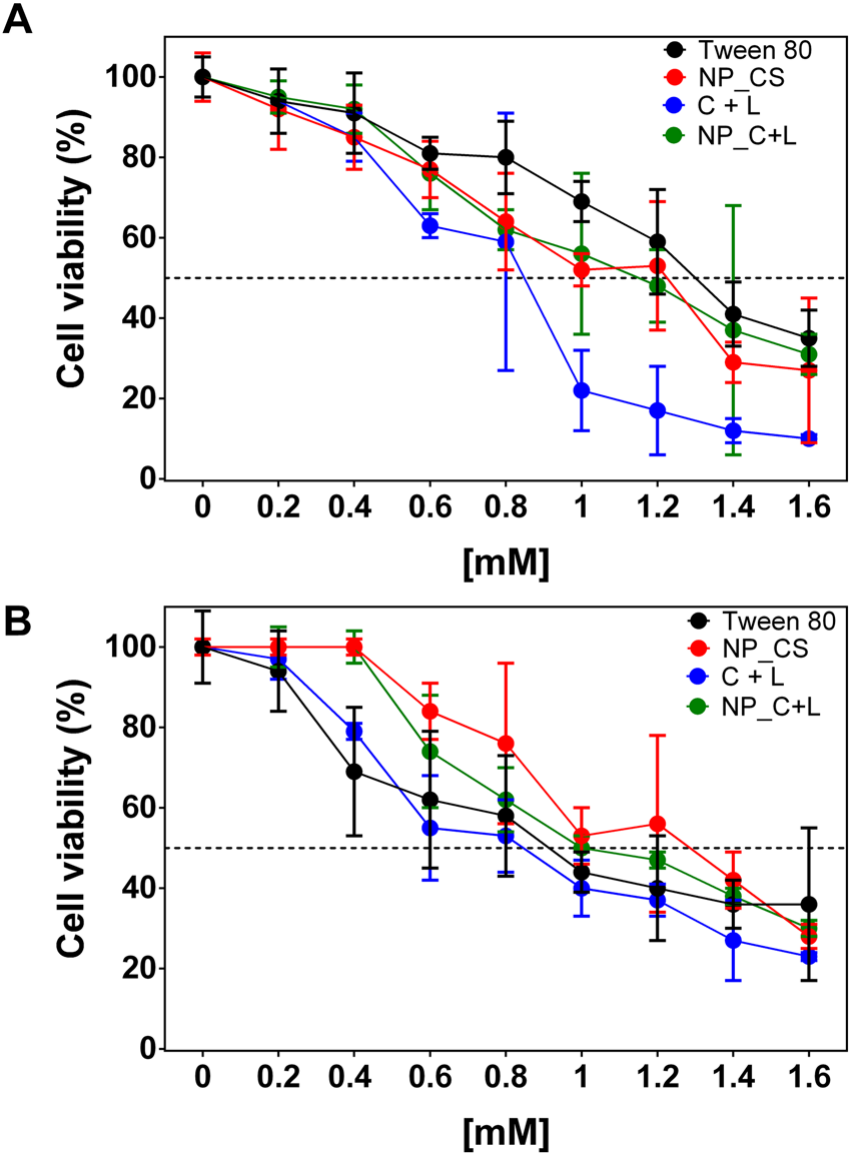
Cytotoxicity assay of the formulations in fibroblasts 3T3 (**A**) and lung cells (V79) (**B**). Cells were treated with the different formulations Tween 80, control nanoparticles (NP), CVC and LNL emulsified (C + L), and nanoparticles containing CVC and LNL co-loaded (NP_C + L) for a period of 24 hours at 37 °C. Cell viability was assessed by the MTT assay, with the control group being considered 100% of cell viability. Values represent the mean ± SD of three determinations (n = 9).

Cell viability assays performed *in vitro* were used to evaluate the cytotoxicities of the nanoencapsulated or emulsified bioactive agents, as well as the empty nanoparticles (Fig. 3). For the control nanoparticles (NP), no significant differences were observed between the cell lines (3T3 and V79), at the different concentrations tested. At the highest concentration (1.6 mM) the cell viabilities obtained for NP were 27% and 28% for the 3T3 (Fig. 3A) and V79 (Fig. 3B) cell lines, respectively. The formulation containing CVC and LNL (NP_C + L) was more toxic towards the V79 cell line, compared to the 3T3 cell line, with IC_50_ values of 1.0 mM and 1.2 mM, respectively. For both cell lines, encapsulation of the compounds resulted in lower cytotoxicity, compared to the emulsified compounds, with the greatest protective effect found for the 3T3 cell line, where the reduction of toxicity was significant from 1 mM. At the highest concentration tested (1.6 mM), the emulsion presented cell viability of 10 ± 1%, while for the nanoparticles containing the bioactive agents the cell viability was 31 ± 5%. These results demonstrated that the nanoencapsulation of the active compounds provided a protective effect against the toxicity of the oils.

Similar results were obtained by Chen *et al*.^58^, who found a significant decrease in the toxicity of carvacrol and eugenol towards the 3T3 cell line when the compounds were encapsulated in chitosan nanoparticles. Kiani *et al*.^52^ observed a 4-fold increase in the viability of gastric adenocarcinoma cells when carvacrol was encapsulated in albumen nanoparticles. Natrajan *et al*.^59^ prepared alginate/chitosan nanoparticles for the encapsulation of turmeric oil and lemongrass oil and evaluated the effect of the nanoencapsulated essential oils on the proliferation of human pulmonary adenocarcinoma epithelial cells (A549). The turmeric oil presented a greater ability to inhibit proliferation of the A549 cells, compared to the lemongrass oil. At concentrations of 0.4 mg/mL, the turmeric and lemongrass oils decreased the cell viability by 40 and 31%, respectively.

### Phytotoxicity assays

The possible phytotoxicity of the nanoparticles was evaluated using pre- and post-emergence treatment of maize (*Z*. *mays*). For all treatments, germination percentages higher than 95% were observed at the highest bioactive agent concentration tested (2.5 mg/mL), with the exception of the emulsified compounds, for which a germination percentage of 76.6 ± 5.7% was obtained. The nanoparticles acted to decrease the effect of the compounds on seed germination.

Table 3 shows the lengths of the shoots and roots of the maize seedlings treated with the nanoformulations containing the compounds at different concentrations (0.05, 0.25, and 2.5 mg/mL). For all the treatments and all the concentrations tested, there were no significant differences in the shoot lengths, with the exception of the emulsified formulation (C + L) at a concentration of 0.25 mg/mL, where there was a significant decrease of the shoot length. In the case of the root length, no significant phytotoxic effects were observed for any of the treatments at concentrations of 2.5 and 0.25 mg/mL. However, at the lowest concentration tested (0.05 mg/mL), there were significant decreases in root length for the seedlings treated with the control nanoparticles (NP_CS) and the emulsified compounds (C + L). The roots are responsible for the absorption of water and nutrients from the soil and their transport to the top of the plant^60^. In addition, the roots are responsible for supplying hormones associated with plant growth and development, as well as for providing mechanical support^60^. The results indicated that the nanoformulations containing CVC and LNL could be used at a concentration of up to 2.5 mg/mL, without inducing phytotoxic effects in the plants.

**Table 3:** Effects of the different formulations on root length, shoot length, dry mass, chlorophyll A, chlorophyll B, and carotenoids of maize plants (*Z*. *mays*) after pre- and post-emergence treatment with surfactant solution (Tween 80), the control nanoparticles (NP), the emulsified CVC and LNL (C + L), and nanoparticles containing CVC and LNL (NP_C + L).

Formulation	Concentration	Parameters					
		Shoot length	Root length	Dry mass	Chlorophyll A	Chlorophyll B	Carotenoids
**PRE-EMERGENCE TREATMENT**							
Tween	0.05 mg/mL	0	0	0	+++↑	++↑	++↑
	0.25 mg/mL	0	0	0	0	0	0
	2.5 mg/mL	0	0	0	++↑	0	0
NP_CS	0.05 mg/mL	0	++↓	0	0	+↑	++↑
	0.25 mg/mL	0	0	0	+↑	+↑	+++↑
	2.5 mg/mL	0	0	0	++↑	+↑	+++↑
C + L	0.05 mg/mL	0	++↓	0	0	0	+↑
	0.25 mg/mL	++↓	0	0	++↑	+↑	+++↑
	2.5 mg/mL	0	0	0	+↑	+++↑	‘++↑
NP_C + L	0.05 mg/mL	0	0	0	++↑	+↑	+++↓
	0.25 mg/mL	0	0	0	0	++↑	+↑
	2.5 mg/mL	0	0	0	0	0	++↑
**POST-EMERGENCE TREATMENT**							
Tween	0.05 mg/mL	0	++↑	0	0	++↑	0
	0.25 mg/mL	0	0	0	0	++↑	0
	2.5 mg/mL	0	+++↑	0	++↑	++↑	++↑
NP_CS	0.05 mg/mL	0	+++↑	0	0	0	0
	0.25 mg/mL	0	+↑	0	0	0	0
	2.5 mg/mL	+++↓	++↑	0	0	0	+++↑
C + L	0.05 mg/mL	0	0	0	++↑	0	++↑
	0.25 mg/mL	+↓	++↑	0	0	0	0
	2.5 mg/mL	+↓	++↑	0	++↑	0	0
NP_C + L	0.05 mg/mL	0	0	0	0	0	0
	0.25 mg/mL	0	++↑	0	0	++↑	0
	2.5 mg/mL	++↓	+++↑	0	+++↑	+++↑	0

A significance level of p < 0.05 was considered for the differences between groups with the same concentration, where + indicates a mild effect, ++ a moderate effect, and +++ a severe effect, relative to the control.

Phytotoxic effects were also evaluated from the photosynthetic activity, with measurements of chlorophyll A, chlorophyll B, and carotenoids, as well as the dry mass of the seedlings. At the higher concentrations tested (2.5 and 0.25 mg/mL), there were significant increases of Chl A (Table 3) for the seedlings treated with surfactant (Tween), the control nanoparticles (NP_CS), and the emulsified compounds (C + L). However, for the nanoparticles containing CVC and LNL, significant increases of Chl A were only observed for the lowest concentration tested (0.05 mg/mL). For Chl B (Table 3), significant increases were observed in the production of this pigment, relative to the control, for the control nanoparticles (NP_CS) at the three concentrations tested, the emulsified compounds at the two highest concentrations (2.5 and 0.25 mg/mL), the nanoparticles at the two lowest concentrations (0.25 and 0.05 mg/mL), and Tween 80 at the lowest concentration tested (0.05 mg/mL). In the case of the carotenoids (Table 3), significant increases of these pigments were observed for all the treatments and all the concentrations, with the exception of Tween 80 at the highest concentration tested (2.5 mg/mL).

Although the plant as a whole can be analyzed to identify possible toxic effects caused by nanoparticles, the leaves are an especially sensitive part of the plant that can provide indications of toxicity by means of symptoms such as chlorosis^61,62^. In the present case, it was notable that the nanoparticles did not present phytotoxicity, since no decreases were observed for the photosynthetic pigments, which would be indicative of chlorosis. Similar results were reported by Choudhary *et al*.^63^, who observed significant increases in Chl A and Chl B in maize plants treated with chitosan nanoparticles. Further evidence that the formulations were not toxic towards the maize plants was the absence of any significant differences in dry mass, relative to the control (Table 3).

For the post-emergence treatments, the germination percentages exceeded 96.6 ± 5.7% in all cases. Significant decreases in shoot length, compared to the control, were found for seedlings treated with the control nanoparticles (NP_CS), nanoparticles containing CVC and LNL at the highest concentration tested (2.5 mg/mL), and the emulsified compounds (C + L) at concentrations of 2.5 and 0.25 mg/mL (Table 3). The greatest effects of the formulations were observed for the root length, which increased. Treatment with the control nanoparticles (NP_CS) resulted in significant increases of root length, relative to the control, for all the concentrations tested, while this effect was observed for treatment with the nanoparticles containing CVC and LNL at concentrations of 0.25 and 0.05 mg/mL. A significant increase in root length, relative to the control, was also observed for the seedlings treated with surfactant (Tween 80) at the highest and lowest concentrations, and for treatment with the emulsified compounds (C + L) at the two highest concentrations tested.

The post-emergence treatments caused smaller effects on the photosynthetic pigments, compared to the post-emergence treatments. For Chl A (Table 3), at the highest concentration tested (2.5 mg/mL) significant increases were observed for the surfactant, the emulsified compounds, and the nanoparticles containing CVC and LNL. A significant increase of this pigment was also observed for the seedlings treated with the emulsified compounds at a concentration of 0.05 mg/mL. In the case of chlorophyll B (Table 3), significant increases of the pigment were only observed for the plants treated with the surfactant at the three concentrations tested and with the nanoparticles containing CVC and LNL at concentrations of 2.5 and 0.25 mg/mL. Significant increases in carotenoids (Table 3) were observed for the surfactant at a concentration of 2.5 mg/mL and for the control nanoparticles, while the emulsified compounds only caused a significant increase of the carotenoids at a concentration of 0.05 mg/mL. As found for the pre-emergence treatments, the post-emergence treatments had no significant effects on the dry mass of the seedlings, relative to the control (Table 3). However, it should be noted that the pre-emergence treatment resulted in greater plant growth (phytomass production), compared to the post-emergence treatment.

Although botanical pesticides are produced by plants, these compounds are not concentrated near the centers of metabolic activity of plant cells, since they are toxic to a wide range of organisms, including the plants that synthesize them^64^. Kordali *et al*.^65^ found high phytotoxicity of the essential oil of *Origanum acutidens* (Hand.-Mazz) and its constituents carvacrol and thymol in the germination and growth of the species *Amaranthus retroflexus* L., *Chenopodium album* L., and *Rumex crispus* L. Pinheiro *et al*.^66^ evaluated the phytotoxic effects of carvacrol, thymol, and the essential oil of *Plectranthus amboinicus* (Lour.) Spreng on the germination and root growth of *Lactuca sativa* L. and *Sorghum bicolor* (L.) Moech. There were concentration-dependent decreases of germination for all the substances tested, for both species, and significant reductions in the shoot and root lengths of both species were observed, compared to the negative control. All the treatments caused significant changes in the cell cycle of the meristematic cells of *L*. *sativa*, with chromosomal alterations observed from the lowest concentrations tested. Synowiec *et al*.^67^ evaluated the effects of six oils on the germination of the agricultural crops *Avena sativa L*., *Brassica napus* L., and *Z*. *mays*. Among the species tested, *Z*. *mays* was the least susceptible to the essential oils and was the only species capable of germinating even following exposure to the highest oil concentrations. Ootani^68^ reported strong toxic effects of citronella and eucalyptus essential oils, as well as citronellal, on the germination and growth of maize (*Z*. *mays*) seedlings, with significant reductions of shoot development and phytomass, compared to the control.

In summary, many of the studies cited above have found negative allelopathic effects associated with the application of botanical pesticides to plants, whether weeds or intended for food production. However, it should be stressed that in most cases, these studies were performed under laboratory conditions using bioassays performed with filter paper in Petri dishes. These types of experiments only consider the response of the plant to a particular treatment. Some studies have already shown that the presence of soil microorganisms plays a fundamental role in reducing the stress/toxicity caused by allelochemicals^69,70^. Hence, in the present work, the exposure of maize plants led to negative effects that were less intense than reported previously, because here the treatments were carried out under field conditions. Furthermore, it is important to highlight that the strategy of encapsulating the compounds in nanoparticles provides a means of protecting them against premature degradation, so that they maintain the concentrations required to control agricultural pests. At the same time, encapsulation acts to protect plants and soil microbiota against the toxic effects of the compounds, since they are released in a sustained manner over a longer period of time.

### Bioactivity assays

#### *Helicoverpa armigera*

The effects of the formulations on the larvae were evaluated considering the mortality rates and the masses of larvae and pupae (sublethal effects) fed with artificial diets treated with the formulations. All the treatments resulted in significantly different mortality rates, relative to the control (Table 4). However, satisfactory mortality was considered to be at least 80%, which was only achieved for the nanoparticles containing CVC and LNL (86 ± 4% mortality). The sublethal effects of the emulsified (C + L) and nanoencapsulated (NP_C + L) compounds were evaluated using the mass of live larvae. Both treatments significantly reduced the larvae mass, with the nanoparticles containing CVC and LNL being most effective. Another interesting feature was that nanoparticles containing only one of the active compounds (data not shown) satisfactorily reduced the larvae population by at least 80%, but in the case of the nanoparticles containing only linalool, there was no significant difference in the residual effect, compared to the control. This revealed the low residual effect of this compound, which has a higher vapor pressure and undergoes faster release, compared to carvacrol. In addition, the nanoparticles containing the two active agents had half the concentration of each compound, compared to the nanoparticles containing only one of the compounds. Therefore, the combination of carvacrol and linalool in the same nanoparticulate system provided enhanced control of *H*. *armigera*, since better effects were observed for larvae treated with this formulation.

**Table 4:** Biological effects of emulsified (C + L) and nanoencapsulated (NP_C + L) CVC and LNL on the mortality and mass of larvae and pupae of *H*. *armigera* fed with artificial diets in the laboratory at 25 ± 1 °C, 70 ± 10% relative humidity, and 14-hour photoperiod.

Formulation	Mortality (%)	Larvae mass (mg)	Pupae mass (mg)
Control	8.0 ± 2.8	0.361 ± 0.065	0.280 ± 0.009
C + L	78.0 ± 3.7^a^	0.096 ± 0.031^a^	0.250 ± 0.0001^a^
NP_C + L	86.0 ± 4.0^a,b^	0.065 ± 0.010^a,b^	0.170 ± 0.004^a,b^

A significance level of p < 0.05 was considered for the differences between the groups, where ^a^represents a significant difference relative to the control and ^b^indicates a significant difference relative to the emulsified compounds.

Besides to reduce the pest population, the chitosan nanoparticles containing CVC and LNL provided a residual effect, negatively affecting the development of the survival larvae and pupae. It could therefore be concluded that the nanoparticles were effective not only in reducing the larvae population, but also in providing a residual effect on the surviving larvae, so that their development was restricted and their negative effects on crops would consequently be diminished. The synergism achieved by the combination of active agents was shown to favor pest control, enhancing the effects of the compounds without requiring their concentrations to be increased.

The combination of different types of essential oils or of different compounds that make up the same oil can provide synergistic or antagonistic activity, enabling optimization or minimization of the effect of the formulation, depending on the mixture. There have been several studies aimed at understanding the nature of the interactions of different compounds that can improve the effectiveness of formulations and consequently reduce the concentrations of the compounds required to achieve satisfactory pest control.

Singh *et al*.^71^ evaluated the effects of eight compounds, individually or in combination: carvacrol, linalool, eugenol, methyl eugenol, 1,8-cineole, α-terpineol, thymol, and trans-anethole. The formulations were applied topically to the third instar larvae of *Chilo partellus* (Swinhoe) (stalk borer). Among the compounds tested, thymol was the most effective (LD_50_ = 189.7 μg/larva), while methyl eugenol was the least effective (LD_50_ = 1069.4 μg/larva). In the case of the mixtures, both thymol and α-terpineol showed synergistic effects in the control of this species when associated with linalool and 1,8-cineol. However, the mixture of the latter two compounds only showed an additive effect in the control of *C*. *partellus*. Using topical application as well as fumigation Pavela^72^, evaluated the effects of six monoterpenes and their mixtures against *Spodoptera littoralis* (Boisduval) larvae. For topical application, thymol and cavacrol were the most effective, with LD_90_ of <100 μg/larva, while γ-terpinene, p-cymene, and 1,8-cineol were the most effective by fumigation (LD_90_ of <100 μg/larva). Among the 15 combinations of compounds tested, using both topical application and fumigation, nine combinations presented synergistic effects, with the most effective being mixtures of p-cymene with γ-terpinene and carvacrol (topical route), α-terpinene with carvacrol, and p-cymene with thymol or carvacrol. None of the combinations tested in this study showed an antagonistic effect.

Lima *et al*.^54^ evaluated the toxicity of the essential oil of *Lippia sidoides* and the compounds carvacrol, 1,8-cineol, and thymol in binary and tertiary mixtures, using fumigation of *Tenebrio molitor*. The greatest toxicity was shown by carvacrol, followed by 1,8-cineol, the essential oil of *L*. *sidoides*, and thymol. All the mixtures presented synergistic effects, except the combination of carvacrol and 1,8-cineol, which had an antagonistic effect (after 48 hours). The mixtures containing thymol showed the most pronounced synergistic effects, evidencing the importance of this compound in synergistic interactions for the control of this species.

Antagonistic effects associated with carvacrol were also observed by Koul *et al*.^73^, who evaluated the effects of five compounds and their binary mixtures on *H*. *armigera*, *S*. *litura*, and *C*. *partellus*. Among the substances tested, thymol was the most effective against these three species, with the most susceptible being *S*. *litura*, while *H*. *armigera* was the least susceptible and the other compounds tested had different effects on the different species. The combination of thymol and linalool presented synergism for all the species tested, whereas carvacrol associated with any of the other substances (linalool, 1,8-cineol, thymol, and trans-anethole) presented antagonistic effects for all three species. In the present work, the mixture of carvacrol with linalool (both emulsified and nanoencapsulated) showed higher activity against *H*. *armigera* following dietary administration, compared to use of the individual compounds, in terms of percentage mortality and the mass of the pupae. In contrast Koul *et al*.^73^, observed an antagonistic effect on the same species when carvacrol was administered in mixtures with any other substance, including linalool. These differences in activity found in different studies can be explained by the type of test employed and interactions between the active agents and both plants and target organisms^33,74^.

#### *Tetranychus urticae*

**Figure 4:**
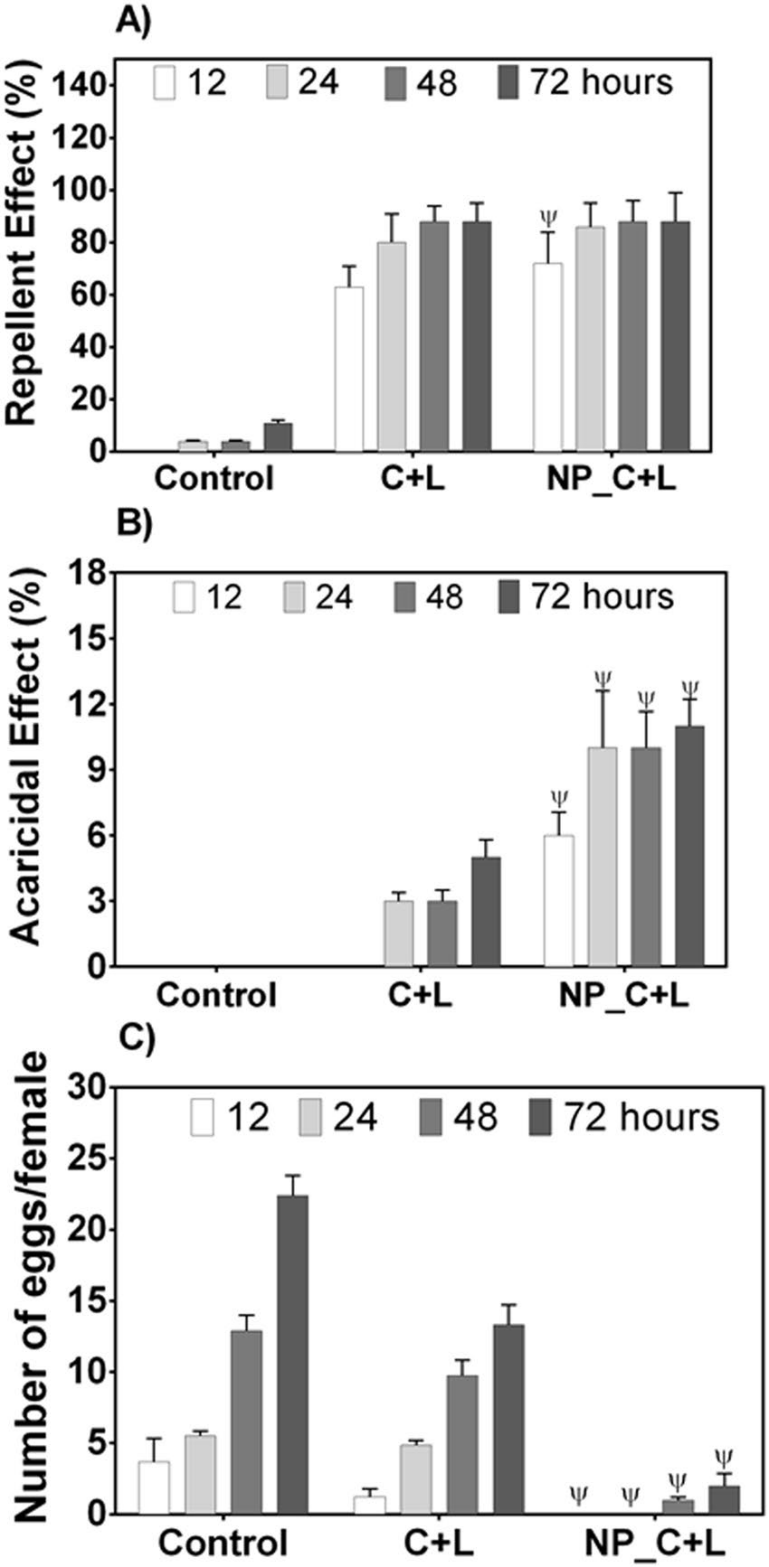
Biological effects of emulsified and nanoencapsulated CVC and LNL against the *T*. *urticae* mite, as a function of time: repellent activity (**A**), acaricidal activity (**B**), and effect on oviposition (**C**). A significance level of p < 0.05 was considered for the differences between the groups, where ψ indicates a significant difference relative to the emulsified compounds.

The results of the repellency assays (Fig. 4A) revealed no significant differences between the effects of the emulsified and nanoencapsulated compounds for any of the times evaluated, except after 12 hours, when the nanoparticles containing CVC and LNL showed a significantly higher (1.14-fold) repellency of the mites, compared to the emulsified compounds. In both cases, the repellent activity increased as a function of time, with the repellency percentage exceeding 80% after 72 hours. In the case of acaricidal activity (Fig. 4B), use of the nanoencapsulated compounds resulted in a significantly higher mite death rate, compared to the emulsified compounds, after all the times evaluated. After 72 hours, use of the emulsified compounds resulted in a mortality percentage (5.0 ± 0.8%) that was less than half that observed for the nanoencapsulated compounds (11 ± 1.22%) after the same period of time. A significant reduction in oviposition of the mites was also observed when the compounds were nanoencapsulated, with a six-fold higher inhibition of oviposition after 72 hours for mites treated with the nanoparticles, compared to those treated with the emulsion. It could be concluded from these results that the nanoparticles containing CVC and LNL were more effective in controlling the mite, and that the control was mainly due to mortality and inhibition of oviposition, rather than repellency. The greater effectiveness of the nanoparticles could be explained by the protection of the active agents against rapid volatilization, as well as their sustained release. It should be highlighted that the nanoparticles containing both compounds were more effective in controlling the mite, considering the three parameters analyzed, compared to nanoparticles containing only one of the compounds (data not shown). The greater effectiveness of the mixed formulations was due to the simultaneous action of the active agents. Linalool was released faster from the nanoparticles and was responsible for the initial effect, while carvacrol was released more slowly, due to its lower vapor pressure, and was responsible for longer-term effects. In addition, carvacrol exhibits mainly insecticidal activity, while linalool exhibits mainly repellent activity.

Wu *et al*.^37^ investigated the acaricidal activity of the essential oil of thyme and mixtures of its major components against mites [*Tetranychus cinnabarinus* (Boisduval)] using sliding immersion bioassays. A greater acaricidal effect was observed for thyme oil, compared to the isolated compounds and their mixtures. Among the isolated compounds, thymol was the most effective. In the case of the mixtures, the association of thymol with linalool or terpinene showed a synergistic acaricidal effect, while a mixture of linalool and carvacrol presented an antagonistic effect. Sertkaya *et al*.^75^ also evaluated the acaricidal effects of four essential oils (thyme, oregano, lavender, and mint) on adults of *T*. *cinnabarinus*. At different concentrations, all the oils caused complete mortality of the mites and did not show phytotoxicity towards the host plant, with the oils of thyme and oregano presenting more pronounced acaricidal activity than the lavender and mint oils. Cavalcanti *et al*.^76^ investigated the acaricidal activity of *L*. *sidoides* essential oil and their constituents (thymol, carvacrol, p-cymene and β-caryophyllene) against *T*. *urticae*. In this study was not observed significant differences in the acaricidal activity between the essential oil of L. sidoides, thymol, carvacrol and β-caryophyllene while p-cymene showed the weaker acaricidal activity among all tested compounds.

## Conclusion

The nanoparticles were effective in control of the two species used in this study, presenting both repellent and insecticidal activity in both cases, while decreased oviposition was also observed for *T*. *urticae*. An important feature was that in both cases, the combined encapsulation of the monoterpenes (carvacrol and linalool) provided greater effectiveness, compared to encapsulation of only one of the compounds (data not shown). However, other studies reported in the literature have found antagonistic effects of mixtures of carvacrol and linalool tested on different types of pests, with decreased effects when compared with use of the individual compounds. This suggests that the encapsulation of the compounds in the chitosan nanoparticles resulted in their sustained release, so that their effects could have occurred at different times, or that the concentrations of the compounds were below the levels at which antagonistic effects could have occurred, resulting in better control of the pests tested. Therefore, the formulations produced in this work could potentially be used as effective alternative methods of pest control, contributing to sustainable agricultural practices.
